# The Contagion of Sentiments during the COVID-19 Pandemic Crisis: The Case of Isolation in Spain

**DOI:** 10.3390/ijerph17165918

**Published:** 2020-08-14

**Authors:** Patricia P. Iglesias-Sánchez, Gustavo Fabián Vaccaro Witt, Francisco E. Cabrera, Carmen Jambrino-Maldonado

**Affiliations:** 1 Department of Economic and Business Administration, University of Malaga, 29071 Málaga, Spain; patricia.iglesias@uma.es; 2 Department of Languages and Computer Sciences. Biomedical Research Institute (IBIMA), Applied Social Research Centre (CISA), University of Malaga, 29071 Málaga, Spain; fabianvaccaro@uma.es (G.F.V.W.); fecabrera@uma.es (F.E.C.)

**Keywords:** sentiment analysis, health crisis, isolation, confinement, emergency response, contagion of emotions, COVID-19

## Abstract

This study examines how confinement measures established during the COVID-19 pandemic crisis affected the emotions of the population. For this purpose, public sentiment on social media and digital ecosystems in Spain is analyzed. We identified affective tones towards media and citizens published on social media focusing on six basic emotions: anger, fear, joy, sadness, disgust and uncertainty. The main contribution of this work is the evidence of contagious sentiments and, consequently, the possibility of using this new dimension of social media as a form of a “collective therapy”. This paper contributes to understanding the impact of confinement measures in a pandemic from the point of view of emotional health. This analysis provides a set of practical implications that can guide conceptual and empirical work in health crisis management with an alternative approach, especially useful for decision-making processes facing emergency responses and health crises, even in an unprecedented global health crisis such as the traumatic events caused by the COVID-19 disease.

## 1. Introduction

Undoubtedly, the world is dealing with a health crisis unparalleled in this era that underscores a negative side of globalization. The World Health Organization (WHO) declared the COVID-19 global pandemic in March 2020. Although this kind of respiratory disease had already caused both widespread death and cases of infections in Wuhan (China) since 2019, the health crisis did not show its true dimensions in Europe up until February and March 2020. From then on, the race to combat the virus escalated worldwide. Spain was one of the countries most affected by COVID-19, establishing a nationwide state of alarm on March 14, 2020. Consequently, the social distancing, self-isolation and quarantine measures in Spain were radical and without precedent; such as the strict stay-at-home instruction for most of the citizens lasting almost two months, and ending with a progressive de-confinement process starting in the first week of May 2020.

During this confinement period, Spanish residents relied on digital ecosystems for disseminating and sharing information from official sources such as the government and health officials; and from the media and other peers. Rodin et al. compiled a list of information producers in the case of a public health crisis that includes international and national public health organizations, national governments, non-governmental organizations, activist groups, news media and citizens [1]. Furthermore, social media plays a significant role in the lives of many of the people affected by the current COVID-19 phenomenon.

Social media has brought opportunities and challenges in health crisis communication, undermining the monopoly of traditional news media [2]. On one hand, social media provide real-time information that combines many types of content, such as text, audio and video. Likewise, the real challenge for organizations is not to provide information in crises like this pandemic, they should also provide a response to audiences; an even greater defiance with the incorporation of social media [3]. Consequently, modern digital ecosystems, including social media, seem to be good platforms to disseminate messages for protective action, for the health and safety of affected publics in health crises [4] and to know what and how the population is feeling in the face of an outbreak like COVID-19.

On the other hand, citizens demand updated and trustworthy reliable information; but, what is more important, they reserve the capacity to generate their own information, even disseminating their personal experiences, speculations, feelings and concerns [5,6]. The previous literature on health crisis communication focused on the effect of response strategies adopted by organizations on publics and, how they acted as result of it [7]; but scarce attention is given to assess the affective pulse of the crisis. In contrast, previous research suggested that emotions might be contagious in social media and how generated content expresses feelings and how other people react to them [8,9], going as far as creating opinion climates through mass and interpersonal communication [10].

Thus, this research work explores the phenomenon of emotion contagion through social media during the COVID-19 confinement stage as a public health issue. Various effects of emotional distress have been related to public health concerns, such as an increase in alcohol consumption and drug abuse [11], eating disorders [12], anxiety and insomnia [13], amongst others. Furthermore, people that who felt strong negative emotions are more likely to accept anger inducing rumors [14]; thus, reinforcing, through feedback, the emotional effects. Within this context, recent research has proposed ways to reduce the stress of the population during social isolation with promising results [15]. However, to the best of the authors’ knowledge, there are no profound studies about the emotional impact produced by the COVID-19 social distancing reported in the literature.

The aim of this study is to assess the emotional impact produced by social distancing, lockdown and isolation of the population in Spain during the COVID-19 global pandemic, expressed in social media and digital ecosystems. This research responds to the lack of research on the intersection of emotional responses during crisis situations and, particularly to those related to public health and social media [5]. Moreover, the findings of this study provide valuable insights based on the repository of emotional content of a traumatic event such as COVID-19.

## 2. Materials and Methods

### 2.1. Study Population and Data Collection

The written opinions related to the social distancing, self-isolation and quarantine measures in Spain were obtained directly from the main media and digital ecosystems: Twitter, YouTube, Instagram, official press websites and Internet forums. The determination of the study universe is complex, because the total population of Spain is 47,100,396 inhabitants, according to the National Institute of Statistics of Spain (INE) data (forecast on July 1, 2019) [16]. Of this population, it is estimated that, on average, 68% are Internet users and that they use social networks and digital media. In principle, each individual Internet user in Spain has the same chances of being included in the study. In order to overcome these difficulties, this study considered communications that expressed unsolicited opinions such as:

- The communication makes explicit reference to social distancing, self-isolation and quarantine measures in Spain.
- The author of the communication is over 18 years old, reported to the services and data sources if available.
- The communication is public and can be viewed without the need for subscription to the data source or explicit permission from the sender of the communication.
- The communication does not come from an advertising campaign.
- The communication has not been generated by automatic procedural methods (bots, fake posts, among others).

The data was collected in the period from March 9 to May 1, 2020; thus, covering the beginning, peak and early de-confinement stages of the COVID-19 related measures in Spain. The collected communications were anonymized, eliminating authorship data, location, source, images, hyperlinks and non-textual components, to leave only the text corpus.

The corpus of communications was analyzed using the natural language analysis tools (ANL) provided by the IBM Watson Analytics service [17]. Then, the corpus was analyzed again for confirmation purposes using the Interval Majority Aggregation Operator (ISMA-OWA) [18], which is designed for decision making in social media with consistent data, leveraged by the combination of computational intelligence and big data techniques [19].

The objective of the natural language analysis was to represent the subjective emotional response of the population as an objective and quantitative time series. The emotional response time series obtained in this way were: anger, disgust, joy, fear, sadness and uncertainty.

### 2.2. Experimental Design

The study period ranged from March 9 to May 1, 2020; and was divided into three distinctive stages.

Stage 1 ranged from March 9 to March 28. This stage covered the events starting with the announcement of the suspension of school activities in the Basque and Madrid autonomous communities of Spain, flight cancellations from Italy, nationwide suspension of university activities, the decree of the national state of alarm and the progressive temporary closure of non-essential businesses.

Stage 2 ranged from March 29 to April 9. This stage covered the events during the peak of the social distancing, self-isolation and quarantine measures in Spain. Most of the population, as well as non-essential workers remained at home.

Finally, Stage 3 ranged from April 10 to May 1. This stage covered the events after the social distancing peak, including the extension of the state of alarm, the slow but progressive lessening of confinement measurements, some of the non-essential workers returned to their jobs, children were permitted to play outside, as well as asymmetric de-confinement measures, depending on the needs of the Spanish autonomous communities.

We expected to see significant differences in the emotional response of the population between the confinement stages. To test this, we performed a non-parametric Kruskal-Wallis H test, considering the stages as the independent variable and the emotional responses as the dependent variables. The normality of the measurement distribution for all the emotional response variables per stage was verified using Kolmogorov-Smirnov and Shapiro-Wilk tests.

To further improve the qualitative analysis of the quantitative data, we performed a hierarchical linear smooth kernel regression on the emotional response time series, using the Epanechnikov quadratic kernel [20]. This approach helps to reduce the noise inherent to data collected from digital ecosystems and provides a graphical representation of the emotion’s tendency. The first hierarchy level corresponded to the complete study period, while the second hierarchy level corresponded to the stages separately. Finally, we calculated the frequency of the words comprising the complete corpus dataset and determined the emotion polarity (positive or negative) related to them.

All the above-mentioned statistical analyses were executed in IBM SPSS 27 (IBM: Armonk, NY, USA). Furthermore, the computational algorithms and natural language analyses were implemented in Python 3 (Python Software Foundation: Wilmington, DE, USA), using the Natural Language Toolkit (NLTK) [21].

## 3. Results

**Figure 1:**
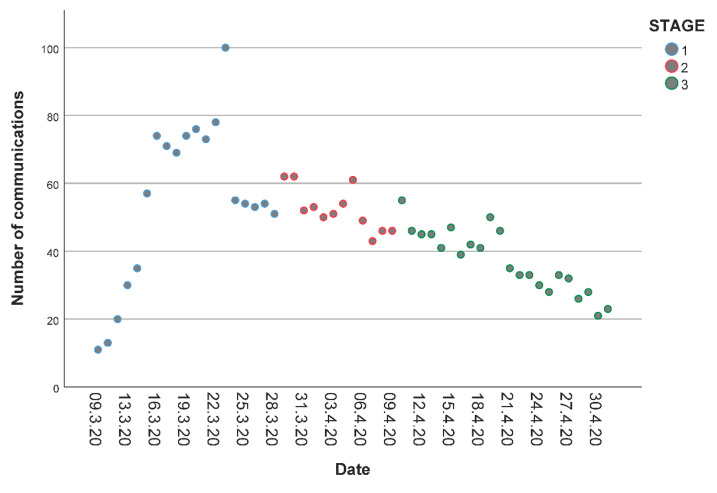
Normalized ratio of communications per day, grouped by stage.

The text corpus contained a total of 80,091 communications that were obtained from Twitter (82.1%), YouTube (12.3%), Instagram (4.2%), official press websites (0.4%) and Internet forums (1.0%). A chart of the normalized distribution of communications per day is shown in Figure 1, where 100 represents the maximum amount of communications obtained in a single day. This figure shows an increasing trend in the number of communications during Stage 1, followed by a progressive decline in the proportion of communications during Stages 2 and 3.

The Kolmogorov-Smirnov and Shapiro-Wilk tests proved the normality of the distributions of all emotional response measurements per stage, with *p* > 0.05 in all cases.

From a generalized point of view, the Kruskal-Wallis H test showed that there was a statistically significant difference in emotional response score related to anger (χ2(2) = 17.806, *p* = 0.000), fear (χ2(2) = 6.462, *p* = 0.040), sadness (χ2(2) = 6.878, *p* = 0.032) and uncertainty (χ2(2) = 7.786, *p* = 0.020). On the other hand, there were no significant differences in the emotional responses related to disgust (χ2(2) = 1.368, *p* = 0.505) and joy (χ2(2) = 3.229, *p* = 0.199).

Due to the inherent differences between social media platforms, we conducted the same analysis on the communications from Twitter, YouTube, Instagram, official press websites and Internet forums separately. These results obtained from Twitter are analogous to the generalized approach, where the Kruskal–Wallis H test showed that there was a statistically significant difference in emotional response score related to anger, fear, sadness and uncertainty (*p* < 0.05); while there were no significant differences in the emotional responses related to disgust and joy (*p* > 0.05). This phenomenon can be traced back to the fact that Twitter provided most of the open-access communications available for this study. In a similar way, the communications obtained from YouTube showed that there was a statistically significant difference in emotional response score related to anger, fear and sadness (*p* < 0.05), but no significant differences in the emotional responses related to uncertainty, disgust and joy (*p* > 0.05). The communications obtained from the Instagram social media platform and online forums only showed statistically significant differences in the emotional response related to anger (*p* < 0.05), but no significant differences in the emotional responses related to fear, sadness, uncertainty, disgust or joy (*p* > 0.05).

On the other hand, the communications obtained from official press websites did not show significant differences in any the emotional response scores measured (*p* > 0.05 in all cases). Due to the small number of communications provided by these sources, it is difficult to establish the causes for this behavior in the emotional response. However, it is important to note that, the nature of official press tends to exhibit a more neutral emotion; therefore, the communications around these topics focus around discussion of facts and less intense emotional opinions.

**Figure 2:**
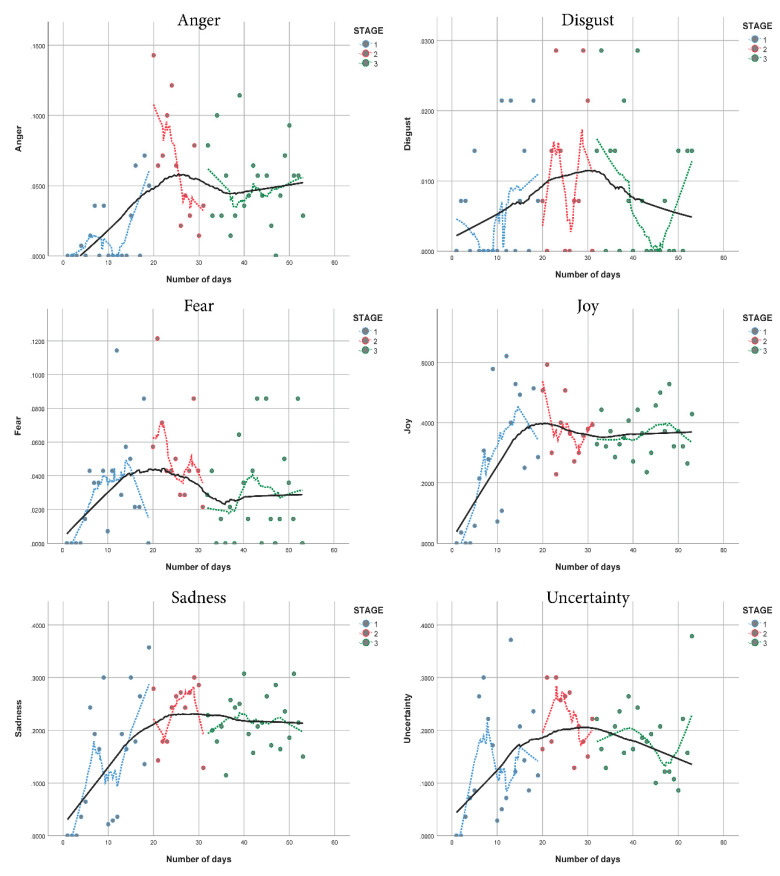
Hierarchical linear smooth kernel regression of the emotional response time series.

The linear smooth kernel regression of the emotional response time series using the Epanechnikov quadratic kernel is shown in Figure 2. The horizontal axis represents the number of days that passed from the start of the study (9 March 2020) and the vertical axis represents the strength of the emotion, normalized from 0 to 1; where 0 is the lowest emotional intensity and 1 is the highest emotional intensity. Moreover, the continuous black line represents the first hierarchical level, and the dashed dotted lines represent the second hierarchical level.

The ratio of communications in isolation increased coinciding with the declaration of the state of alarm in Spain, this increment is slightly faster than that of general communications about the COVID-19 disease.

**Figure 3:**
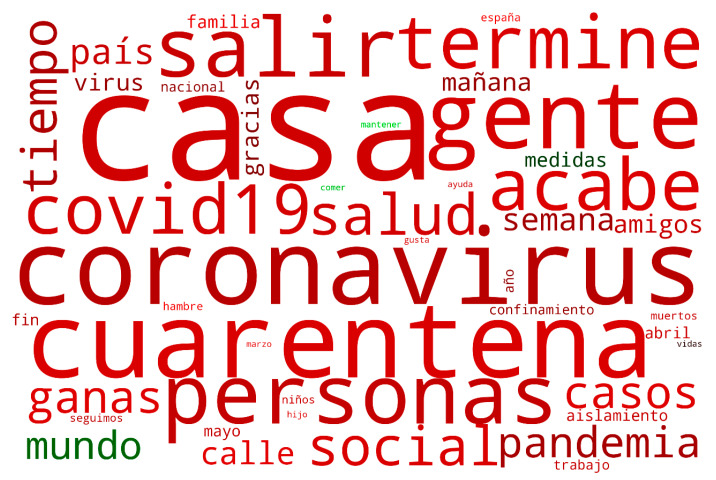
Word-cloud representing the word frequency and emotion polarity.

The frequency of the words and the emotion polarity associated with them is displayed in Figure 3. The frequency ratio between words is expressed as the relative word size. On the other hand, the color represents the polarity in a scale ranging from bright green (absolute positive) to bright red (absolute negative); and where black represents a neutral emotion. The words in the Spanish language that expressed the highest frequency, in other words, the highest presence, are “casa” (house), “coronavirus”, “cuarentena” (quarantine), “gente” (people), “personas” (people), “salir” (go out) and “termine” (end). Moreover, most of the words shown in Figure 3 are displayed in red, thus, representing a mostly negative emotion polarity, with a mean polarity of −0.3557 and a standard deviation of 0.2438. The exceptions to this negative trend are the words “mundo” (world), “medidas” (measures), “comer” (to eat) and “mantener” (to maintain); which showed a neutral-positive trend.

## 4. Discussion

The amount of user-generated content and social-media communications related to the social distancing, self-isolation and quarantine measures in Spain increased rapidly and steadily during Stage 1 of the study period. This coincided with the declaration of the sanitary state of alarm and the first of isolation measurements. A slight first peak in the raw amount of communications during this stage occurred between March 9 and 12, triggered by the suspension of academic activities in Madrid and the announcement of the isolation measurements, respectively. Moreover, the days near the end of the Stage 1, around March 22, reported the highest amount of communications; matching the discussion about the first extension of the state of alarm.

The communications gathered from Stage 2 were focused around two main discussion areas. Firstly, people were talking about the causes and effects of isolation; and secondly, the social media flourished with interesting proposals to cope with the isolation and stress of not being allowed to leave home. On the other hand, there was an increase in the amount of communications criticizing the government-imposed isolation measurements.

Stage 3 was the longest of the three stages, and marked the progressive lessening of the confinement measurements. The communications posted in online ecosystems during Stage 3 showed an increased interest in the economic effects of the confinement in Spain. Then again, social media conversations gradually declined along with the reduction of the confinement measures (Figure 1).

Results suggest that the isolation measures in Spain significantly affected the emotions of the population. This effect is more noticeable through the anger emotional response. The smooth kernel regression of the anger timeline (Figure 2) shows its highest peak around the beginning of Stage 2, roughly around March 29. It is important to notice that results also suggest that the isolation measures did not affect the joy or the disgust emotional responses of the population. The smooth kernel regression of the joy timeline (Figure 2) shows a steady increase during Stage 1 and a horizontal stabilization during Stages 2 and 3.

The results show that all the studied emotional responses have an important presence during the analyzed isolation period; apart from disgust, that registered a minimal presence. The highlights of the emotional responses are detailed in Table 1, alongside the date that is most closely related to each event.

The social media coverage of isolation was extensive, and a broad array of topics were addressed, as shown in Figure 3. However, there are four main topic groups: firstly, those that are specifically focused on the COVID-19 illness, mainly regarding deaths, treatments and infection rates. Secondly, those concerning lockdown, including self-isolation, confinement, home, family and going out. Thirdly, there are topics that show society’s concerns due to the uncertainty of the situation and its consequences, such as money, work, hunger, help and world. Lastly, there were other topics that could not be classified within the first three groups, such as gratitude, donations, personal protection equipment and solidarity. Moreover, it should be noted that, when considering daily usage of Twitter hashtags during the pandemic, some of the most widely used during the study period were #yomequedoencasa (I am staying at home) and #quedatencasa (stay at home) with the intention of urging people to stay at home. The effect of these kinds of conversations in social media can also be appreciated in Figure 3, as the frequency of words such as “cuarentena” (quarantine) and “casa” (home) are very high. Furthermore, this also explains the prevalence of words such as “salir” (go out) “termine” (finish), since people were also discussing their desire for this situation to end.

It should be noted that the emotional response timelines displayed in Figure 2 suggest a pattern of polarization on the topic of isolation. Many communications expressed personal experiences, sentiments and proposals to stay at home, even initiatives to support medical staff and other front-line professionals; especially during the first half of the study period, in other words, near the end of Stage 2. On the other hand, during Stage 3 the focus of the citizens shifted towards the effects of the confinement measures, and the possible negative consequences of the new normality.

Regarding the coexistence of feelings, it should be noted that there are some events that clearly show the synergies of feelings. For example, March 27 marks the beginning of widespread temporary employment regulations in Spain, designed to mitigate the economic consequences of the COVID-19 pandemic. The peaks of joy and sadness reflect the mixed feelings on this issue (Figure 2). Another example of synergism between joy and fear happened on April 26. On this date the government allowed children under 14 years old to play outside. This was an expected measure, but a large portion of the population was worried about a possible new peak in infections. Moreover, during the first days of Stage 1, both anger and fear displayed a slight peak in the second hierarchical level of analysis, in other words, the dotted lines in Figure 2. The death toll seems to be the main cause of this peak; nevertheless, this behavior shifted towards the end of Stage 3, where people expressed their concerns about the new normality.

The analysis of the emotional content in the context of isolation due to COVID-19 provides valuable insights into how sentiments are affected by confinement measures and how they are expressed and spread on social media. Previous literature has addressed this issue by focusing on economic and financial crisis impacts [6] and how media content studies have been applied in health crisis management, which have been observed more from the communication point of view and more specifically through a public relations lens [1,4]. Although some authors have already pointed out that feelings expressed in online conversations can be contagious both in a positive [9,15] and a negative way [11,12,13], this work covers the gap by analyzing the public’s sentiments in digital ecosystems as a health issue. The emotional reactions detected were in line with the general sentiment captured by the analysis of Thelwall et al. [8] and Neubaum and Krämer [10]. The commentaries featured in online conversations highlight the societal impact and health implications of the pandemic as well as their implications in the global health crisis. Additionally, it coincides with Thelwall et al. [8] that sentiment detection in social media is challenging and can provide an interesting insight into facing the crisis, especially for those institutions with competences in this field. In any event, there are a few literature works focused on monitoring sentiments in social media [9,11,12,13,15] but not many through a health lens [5,7] while taking into account the usefulness of analysis in health crisis management by considering all the responsible actors.

This research is limited to the analysis of communications freely available in digital ecosystems; therefore, we consider that further studies using public opinion surveys, which could deepen the understanding of the emotional responses of the population, are needed. Furthermore, this research focused solely on the hardest stages of COVID-19 isolation in Spain; thus, we recommend this study be replicated for other countries.

## 5. Conclusions

The social distancing, self-isolation and lockdown measures in Spain significantly affected the emotions of the population. Furthermore, the driver of these emotions evolved and shifted during the stages of the confinement. The results of this study provide a snapshot of the emotional response of the Spanish population during the COVID-19 isolation. Likewise, a repository of emotional content of an unprecedented outbreak is provided. Furthermore, it reveals that social media can be a form of “social therapy” and a suitable approach for health crisis communication. This research responds to the lack of research at the intersection of emotional responses during crisis situations and, particularly to those related to public health and social media. Moreover, the findings of this study provide valuable insights based on the repository of emotional content of a traumatic event such as the COVID-19. The findings of this research are useful for encouraging health institutions to improve the management of social media and digital ecosystems during a health crisis; and to polish decision-making processes facing emergency responses.

## Figures and Tables

**Table 1:** Emotional response highlights.

Emotion	Highlight Date	COVID-19 Event
Joy	22/03	Deployment of field hospitals, highlighting IFEMA in Madrid to avoid the collapse of the medical system.
	14/04	Some of the countries most affected by COVID-19 experienced a decrease in infection rates, e.g., Germany, and others; that started to implement some measures in order to lessen the impact of isolation in the economy.
	18/04	A new procedure to count the COVID-19 cases was implemented and suggested a possible decline in the number of real cases reported.
	26/04	Children under 14 years of age were allowed to play outside for one hour per day.
Sadness	14/03	The Spanish government declared a national state of alarm and launched an advertising campaign on social media using the hashtag #estevirusloparamosunidos (that means “we will stop this virus together”) Massive applause to recognize the contribution and courage of medical staff.
	26/03	International media harshly criticized the Spanish government for their management of the health crisis.
	10/04	The daily death toll is estimated to be around 700 deceased.
	22/04	Slight increase of daily death toll.
Anger	12/03	The Spanish government considered the declaration of a state of alarm due to the death toll and spread of the virus. Some Spanish regions, such as Catalonia, declared local isolation measures.
	24/03	Death toll reached alarming figures. People were highly concerned about the infection rate.
	14/04	Wuhan added 1290 deceased to official figures, 50% more than first announced.
Disgust	25/03	Spain, with 3434 deceased, exceeded the death toll in China. One of the most lethal days (734 deceased).
	18/04	The death toll in Spain rose again, triggering discussion and discrepancies with the case counting method.
Fear	24/03	The infection rate and death toll had been increasing steadily since March 16, and no changes in this trend were visible.
	26/04	Children under 14 years old were allowed to play outside for one hour per day.
	01/05	Beginning of Phase 1 of the de-escalation process in a large part of the Spanish territory.
